# Injectable antibiotic use in India: public-private share in volume and cost

**DOI:** 10.12688/wellcomeopenres.20633.1

**Published:** 2024-02-19

**Authors:** Shaffi Fazaludeen Koya, Senthil Ganesh, Katherine Klemperer, Prashant Yadav, Anthony McDonnell

**Affiliations:** 1 Boston University School of Public Health, Boston, Massachusetts, 02125, USA; 2 Public Health Foundation of India, New Delhi, Delhi, India; 3 Center for Global Development, London, UK; 4 Center for Global Development, Washington, District of Columbia, USA; 5 INSEAD, Fontainebleau, France

**Keywords:** Antibiotic consumption, defined daily doses, antibiotic stewardship, drug utilization, drug costs.

## Abstract

**Background:**

Consumption of injectable antibiotics is not widely studied, despite injectables constitute a major share of antibiotic cost. This study aimed to understand the share of oral and injectable antibiotic consumption and cost at the national level in India, and the public and private sector shares in the provision and cost of injectables in Kerala state.

**Methods:**

We used the PharmaTrac private sector sales dataset and the Kerala Medical Services Corporation public sector procurement dataset. Using WHO Access, Watch, Reserve (AWaRe) and Anatomical Therapeutic Chemical (ATC) Classifications, we estimated the annual total and per-capita consumption, and the annual total, per defined daily dose (DDD), and per-capita spending on injectables.

**Results:**

Although 94.9% of total antibiotics consumed at the national level were oral preparations, 35.8% of total spending were on injectables. In Kerala , around 33% of total antibiotic spending in the private sector were for injectables, compared to around 25% in the public sector. The public sector used fewer injectable antibiotic formulations (n=21) compared the private sector (n=69). The cost per DDD was significantly higher in the private sector as compared to the public sector. Despite only accounting for 6.3% of the cost share, the public sector provided 31.4% of injectables, indicating very high efficiency. Across both sectors, Watch group antibiotics were significantly more consumed and at a significantly higher cost than Access group antibiotics, for example in nearly double the quantity and at 1.75 times the price per DDD in the private sector. Reserve group antibiotics made up the lowest consumption share (0.61% in the private sector), but at the highest cost per DDD (over 16 times that of Access).

**Conclusions:**

Public sector showed higher cost efficiency in antibiotic provisioning compared to private sector. Appropriate antibiotic use cannot be achieved through drug price control alone but requires extensive engagement with private providers through structured stewardship programs.

## Plain Language Summary

This study tried to understand the share of public and private sectors in the volume and cost of antibiotic injections in India, particularly in the state of Kerala. We used drug sales data (PharmaTrac) and Kerala government procurement data for the analysis. The study was conducted by researchers at Boston University (USA), Public Health Foundation of India (India), Center for Global Development (UK and USA), and INSEAD (France), and was supported by a Wellcome grant. We analysed data using the World Health Organization classification of antibiotics into Access, Watch, Reserve (AWaRe), which is based on the risk of emergence of resistance. We estimated the annual total and per-capita consumption, and the annual total, per-dose, and per-capita spending on injectables. We found that although antibiotic injections were less than six percent of total antibiotics consumed nationally, they accounted for more than 35% of total spending. Kerala data showed that the public sector showed higher efficiency by providing one-third of antibiotic injection doses using fewer formulations, with only six percent of the cost share. Reserve group antibiotics, which made up the lowest consumption share, had the highest cost per dose (over 16 times that of Access antibiotics). In conclusion, public sector showed higher cost efficiency in injectable antibiotic provisioning compared with private sector. Appropriate antibiotic use requires extensive engagement with private providers through structured stewardship programs.

## Introduction

Antimicrobial resistance (AMR), particularly antibiotic resistance, is recognized as one of the top 10 global public health threats facing humanity ^1^. The first World Health Organization (WHO) global surveillance report on antibiotic resistance (ABR) in 2014 showed that five out of the six WHO regions had more than 50% resistance to third generation cephalosporins and fluoroquinolones in Escherichia coli and methicillin resistance in Staphylococcus aureus in hospital settings ^2^. India had the worst drug resistance index (DRI) in 2019—an index that represents burden of antibiotic resistance across multiple pathogen ^3^.

India is the largest consumer of antibiotics in the world by volume, although the country’s per-capita consumption rate is much lower compared to other countries ^4^. There exist wide differences in consumption rates between different states in India, and the states with high infectious disease burden have lower per capita consumption— at least as per private sector consumption data ^5^. Moreover, the country faces serious challenges related to inappropriate use of antibiotics— due to a range of factors including those related to patient expectations, lack of effective regulations or implementation of existing regulations, and market forces.

Anatomical Therapeutic Chemical (ATC) system which classifies drugs according to the human organ or system and their therapeutic, pharmacological, and chemical properties and the WHO’s Access, Watch, Reserve (AWaRe) classification are increasingly used for promoting antibiotic stewardship (AMS) and assessing inappropriate use. Although inappropriate use of any antibiotic is a public health challenge, the inappropriate use or overuse of injectable antibiotics is especially problematic as most of them are newer generation molecules that are classified as Watch (ones that should be used “watchfully”) or Reserve (ones that should be kept “reserved” for patients who truly need them based on culture and sensitivity tests) as opposed to Access. At the same time, there is also a serious issue of lack of access to these lifesaving drugs for a significant proportion of patients in the country due to affordability and availability ^6^.

The direct and indirect costs of AMR are significant, and are not limited to death and disability; reviews have shown that the associated cost for treating the prolonged illness due to drug resistance strains is significant ^7^. Therefore, existing health inequities complicate the problem in the Indian context. To illustrate, more than 60% of healthcare institutions and 80% of doctors in India are concentrated in urban areas – whereas more than 70% population live in rural areas ^8^. Similarly, more than 80% of outpatient consultations and close to 60% hospitalized care including 40% of births happen in private sector ^9^. Globally, national governments spent 6% of GDP in health (year 2019); however, India’s public spending on health remained around 1% of GDP for the last several years— lower than that in many low-income countries ^10^.

Out-of-pocket health expenditure (OOPE) is so high that it pushes around five percent of India’s population into poverty (medical impoverishment) every year ^11, 12^. Cost of drugs constitutes a significant share of these OOPE. For example, data from Consumer Expenditure Surveys and the Social Consumption of Health Survey showed that out-of-pocket medicine costs alone account for an estimated 11% of families experiencing financial catastrophe, defined as at least 10% of overall household consumption expenditure ^13^.

Extant literature discusses consumption patterns of antibiotics in the public sector in Low- and Middle-Income Countries (LMICs). Discussions about the consumption patterns of antibiotics in private pharmacies and hospitals have been more focused on oral community-use antibiotics. There is lack of analysis regarding the consumption patterns of injectable and Reserve category antibiotics in the private sector.

A key step in implementing new payment and procurement models for antibiotics to mitigate the risks of AMR is understanding how antibiotics are used in public and private channels, and how these sectors contribute to overall use and cost. Although community consumption patterns of oral antibiotics have been analyzed in several studies, relatively little is known about injectable antibiotic consumption - which often involves significant out-of-pocket payments at private pharmacies. Injectables are mostly used in inpatient settings, but due to the lack of availability of medicines within hospitals, patients who undergo inpatient treatment in a public facility also end up purchasing these medicines from private pharmacies. Given this background, this research will explore injectable antibiotic use in India (using the state of Kerala for primary analysis) and the associated cost with the following broad objectives.

## Objectives

1. To estimate the annual total volume and per-capita rate of injectable antibiotic consumption in the private sector at the national level, across WHO AWaRe groups and ATC levels

2. To estimate the annual total volume and per-capita rate of injectable antibiotic consumption in the public and private sectors in the state of Kerala, across WHO AWaRe groups and ATC levels

3. To estimate the annual total spending, per dose spending, and per-capita spending on injectable antibiotics in Kerala.

## Methods

Public and Patient Involvement: This study used only drugs sales and procurement data, and did not involve public and patients in any way.

We used Defined Daily Dose (DDD), which is the assumed average maintenance dose per day used for the main indication in adults for a drug based on its ATC groups. We used the DDD values based on the ATC/DDD 2022 index prepared by the WHO Collaborating Centre in Oslo ^14^. Further, to adjust for population size, we calculated DDD per 1,000 population per day (“DID”) at national and state levels. Analysis is conducted using data for the four years, 2016 to 2019, and the results are provided as an average of the four years.

For the national and Kerala state-level private sector analysis, we used a nationally representative private drugs sales dataset, PharmaTrac, from the AIOCD— a national network of pharmaceutical distributors in India. This dataset contains sales volume (number of packages), package size, and product strength besides unit price for each product sold in the market. We used the Kerala Medical Services Corporation Limited (KMSCL) data for the public sector analysis at Kerala state level.

First, we calculated the total DDD consumed per pack using the product and pack size information from the PharmaTrac data and the corresponding formulation’s DDD information from the WHO ATC/DDD index 2019 list ( https://aware.essentialmeds.org/groups). The DDD per pack is then multiplied by the total number of packs (MAT) consumed per year to obtain the total DDD of the product for the whole year, and then the DDD is summed up at the molecule level.

We calculated DDDs using the following formula.

$$\text{Total}\;\text{DDDs}\;\text{Consumed}=\frac{\text{Strength}*\text{Pack}\;\text{Size}*\text{Packs}\;\text{Consumed}}{\text{DDD}\;\text{of}\;\text{Molecule}/\text{formulation}}$$

Second, we used the projected mid-year population obtained from the National Population Commission ( www.censusindia.gov.in) to derive the DDD per 1000 persons per day, a standard unit used in global literature (DID) to measure the per-population consumption rate. We used the projected mid-year population for all the years based on the 2011 census obtained from the National Population Commission for calculations. DID is calculated as follows:

$$\text{DIDs}=\frac{\text{Total}\;\text{DDDs}\;\text{Consumed}}{\text{Population}\;\text{in}\;\text{thousands}*365}$$

Third, we estimated the total cost of these products at the individual molecule level and at the level of antibiotic classes and groups using the maximum retail price (MRP) data available from PharmaTrac. MRP refers to the maximum price at which a product can be sold to the consumer in the private sector pharmacies or hospitals after including the profit margins, and therefore represents the cost incurred by the patients treated at the private sector at the point of care.

Fourth, we estimated the public sector consumption and cost to the government (in Indian Rupees) of injectable antibiotics for the state of Kerala using data from KMSCL. Finally, we calculated the total antibiotic consumption and cost by using data from KMSCL and PharmaTrac. This allowed us to determine the share of public and private sector injectable antibiotic consumption and costs in Kerala. The per capita cost and per dose (per DDD) cost at public and private sector were compared across antibiotic groups. To account for the difference between MRP and the actual procurement price in the public sector—as the public sector purchases medicines in bulk quantity through tender—we conducted a sensitivity analysis. For this, we used the average wholesale price in the private sector instead of MRP to estimate the public - private shares of injectable antibiotic cost. Wholesale price refers to MRP minus the profit margin for pharmacies or hospitals, applying wholesale prices will make the private sector purchase prices closer to public sector procurement price. All analyses used WHO AWaRe categorization and ATC classification to understand how price regulation works towards antibiotic stewardship at the policy level to modulate the consumption of restricted molecules.

## Data analysis

All analyses were performed using Microsoft Excel and R Statistical Software (v4.1.2; R Core Team 2021).

## Results and discussion

### National level consumption

At the national level, the average annual DDD consumed per 1000 population per day was 10.69 of which 94.9% was oral antibiotics. ( Table 1) However, 35.8% of total spending on antibiotics was on injectables, and the cost per DDD for injectable antibiotics was more than 10 times the cost per DDD for oral antibiotics.

**Table 1.** National level private sector consumption and costs of antibiotics, 2016–19.

Route	DDDs consumed, share	Cost ^$^, share	Per capita cost #	Cost per DDD
Injectable	262.6 million 5.1%	75.7 billion, 35.8%	57.7	288.2
Oral	4,860.9 million 94.9%	135.9 billion, 64.2%	103.5	27.9
Total	5,123.5 million 100%	211.6 billion, 100%	161.2	41.3

^$^all costs are in Indian Rupees (₹); #projected population as per 2011 Census— 1.31 billion.

More than 50% of the injectable antibiotics used in the private sector in India belonged to the Watch group (133.7 million DDDs), and 21.9% (57.7 million DDDs) belonged to WHO Discouraged groups of antibiotics. ( Table 2). While Access group antibiotics constituted 26.5% of DDDs, they contributed 15.4% of the total cost on injectables. In comparison, Reserve group antibiotics constituted 5.9% of total cost despite constituting less than 1% of consumption.

**Table 2.** National level consumption and cost of injectable antibiotics in private sector across AWaRe groups, 2016–19.

AWaRe Category	DDDs, share	Cost, ^$^ share	Cost per DDD
Access	69.5 million 26.5%	11.6 billion 15.4%	167.5
Watch	133.7 million 50.9%	39.6 billion 52.3%	296.2
Reserve	1.6 million 0.61%	4.5 billion 5.9%	2,798.8
Discouraged	57.7 million 21.9%	19.9 billion 26.3%	345.0
Not Listed	18,520 0.01%	36.1 million 0.05%	1,951.8
Total	262.6 million 100%	75.7 billion 100%	288.2

^$^all costs are in Indian Rupees (₹).

At the antimicrobial group level (ATC 3 level), cephalosporins and other non-penicillin beta-lactam antibiotics were the most consumed injectables at the national level during 2016-19. ( Table 3) Twice as much aminoglycosides as penicillin were consumed (22.7% vs 11.2% of total DDDs) but at a slightly lower cost (11.2% of total cost vs 12.7%). Tetracyclines (J01A) and other antibacterials (J01X including polymyxins and glycopeptides) have very high per DDD costs (2574.0 and 2223.6 Indian Rupees, respectively), but they make up a very low share of DDDs consumed —0.14% and 0.94% respectively. At the sub-group level, the third generation cephalosporins (J01DD) constituted the majority of injectables consumed (53.2%). ( Table 4) Ceftriaxone was the most consumed injectable (33.6%). At 131.31 INR per DDD, the total cost of ceftriaxone used in the private sector was over 1,150 Crores of Indian Rupees (more than 142 million USD) per year, which constitutes 15.3% of total injectable antibiotic cost. ( Table 5)

**Table 3.** Consumption and cost of top five injectable antibiotic groups in private sector, ATC 3 level, 2016–19.

Antibiotic Group (ATC3)	DDDs, share	Cost, share	Cost per DDD
Other beta-lactam antibacterials, J01D	145.8 million 55.5%	45.7 billion 60.4%	313.8
Aminoglycoside antibacterials, J01G	59.6 million 22.7%	8.4 billion 11.2%	141.7
Beta-lactam antibacterials, penicillin, J01C	29.3 million 11.2%	9.6 billion 12.7%	328.3
Macrolides, lincosamides and streptogramins, J01F	9.5 million 3.6%	1.7 billion 2.3%	182.9
Quinolone antibacterials, J01M	8.5 million 3.2%	1.8 billion 2.4%	213.6
All the other groups	9.9 million 3.8%	8.3 billion 1.0%	1,194.5

^$^all costs in Indian Rupees.

**Table 4.** Consumption and cost of injectable antibiotics across major sub-group level (ATC 4), national level, 2016–19.

Antibiotic Group	DDDs, share	Cost, share	Cost per DDD
Third generation cephalosporins, J01DD	139.7 million 53.2%	32.0 billion 42.3%	229.2
Other aminoglycosides, J01GB	59.6 million 22.7%	8.4 billion 11.2%	141.6
Combinations of penicillin, incl. BLIs, J01CR	24.1 million 9.2%	9.3 billion 12.3%	386.1
Lincosamides, J01FF	8.8 million 3.3%	1.5 billion 2.0%	173.2
Fluoroquinolones, J01MA	8.5 million 3.2%	1.8 billion 2.4%	213.6
All the other groups	21.9 million 8.3%	22.6 billion 29.9%	1,662.8
Total	262.6 million 100%	75.7 billion 100%	288.2

Note: all costs are in Indian Rupees (₹).

**Table 5.** Consumption and cost at antibiotic molecule level (top five), national level, 2016–19.

Antibiotic	DDDs, share	Cost, ^$^ share	Cost per DDD
Ceftriaxone	88.1 million 33.6%	11.6 billion 15.3%	131.3
Amikacin	35.3 million 13.5%	7.3 billion 9.7%	207.3
Gentamicin	22.8 million 8.7%	661.2 million 0.87%	29.0
Ceftriaxone - sulbactam	17.2 million 6.6%	5.7 billion 7.5%	330.7
Cefotaxime	13.8 million 5.3%	2.5 billion 3.2%	177.6
All the other antibiotics	85.3 million 32.4%	48.0 billion 63.4%	1,705.5

Note ^: $^all costs are in Indian Rupees (₹).

### Consumption in Kerala, 2016-2019

The average annual antibiotic use in Kerala during 2016-19 was 285.7 million DDDs (22.4 DDD per 1000 persons per day) of which public sector ( Table 6) contributed 113.0 million (39.5%). By antibiotic form, the public sector represented 39.8% of oral and 31.4% of injectable consumption. However, public sector spending accounted for only 8.3% of total spending on antibiotics.

**Table 6.** Annual volume and share of DDDs, across public and private sector, oral and injectables, Kerala State, India, 2016–19.

	Public DDDs, %	Private DDDs, %	Total	Public Cost, %	Private DDDs, %	Total
Oral	110.4 million, 39.8%	167.2 million, 60.2%	277.6 million	₹ 500.6, 9.3%	₹ 4,909.5, 90.7%	₹ 5,410.1
Injectable	2.6 million, 31.4%	5.5 million, 68.6%	8.1 million	₹ 167.4, 6.3%	₹ 2,505.3, 93.7%	₹ 2,672.7
Total	113.0 million, 39.5%	172.7 million, 60.5%	285.7 million	₹ 668.0, 8.3%	₹ 7,414.8, 91.7%	₹ 8,082.8

Note: all DDDs are in millions; all costs are in million Indian Rupees (₹). The price for public sector is at actual cost in which public sector (KMSCL) purchased medicine. The price for private sector is based on MRP.

**Figure 1 [A-C].**
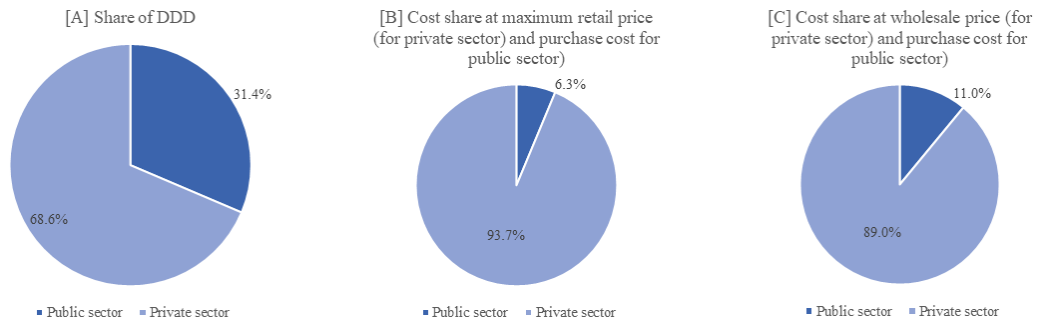
Volume and cost of injectable antibiotics: public-private share in Kerala.

The sensitivity analysis ( Figure 1 A-C) shows that even when calculated using wholesale price, the cost share of injectables in the private sector was disproportionately high relative to the 68.6% volume share. When calculated using MRP, the private sector contributed 93.7% of total cost; when calculated using wholesale price, it contributed 89.0%.

The per-capita expenditure for injectables in the private sector was INR 71.8 compared to INR 4.8 in the public sector. More than 33% of total spending in private sector is for injectables, compared to a little over 25% in public sector ( Table 7).

**Table 7.** Public and private spending in antibiotics in Kerala (average for the period 2016–19).

	Injectable		Oral	
Sector	Total cost, %	Per-capita cost	Total cost, %	Per-capita cost
Private	2,505 million, 33.8%	71.8	4,909 million, 66.2%	140.6
Public	167 million, 25.1%	4.8	500 million, 74.9%	14.3
Total	2,672 million, 33.1%	76.5	5,410 million, 66.9%	154.9

Note: all costs are in Indian Rupees (₹).

In terms of AWaRe groups, while one-third of Access and Watch molecules are provided by the public sector, one-fourth of Reserve and one-fifth of Discouraged injectables are provided by the public sector. ( Table 8). The share of Access DDDs in total consumption was 12.4% (2.7 percentage points) higher in the public sector compared to the private sector and share of Watch DDDs was 9.2% (5.4 percentage points) higher in the public sector compared to the private sector. The share of Reserve DDDs was 21.7% (0.26 percentage points) lower and share of Discouraged was 42.5% (7.6 percentage points) lower in the public sector compared to the respective shares in the private sector. ( Table 9) The total cost and per DDD cost is markedly higher in the private sector compared to the public sector across all AWaRe groups. The cost per DDD for Reserve antibiotics is the highest in both sectors, but the cost of purchase per DDD in the public sector is 93.0% lower compared to the MRP per DDD in the private sector.

**Table 8.** Volume and share of injectables across AWaRe groups—public and private sectors, Kerala state, India.

AWaRe groups	Public *(DDD, (DID), share)*	Private *(DDD, DID, share)*	Total
Access	0.63 million, (0.049), 34.0%	1.2 million, (0.096), 66.0%	1.8 million
Watch	1.6 million, (0.129), 33.3%	3.3 million, (0.259), 66.7%	4.9 million
Reserve	0.02 million, (0.002), 25.8%	0.07million, (0.005), 74.2%	0.09 million
Discouraged	0.26 million, (0.021), 20.8%	1.0 million, (0.079) 79.2%	1.26 million
Total	2.6 million, 31.4%	5.5 million, 68.6%	8.1 million

**Table 9.** Total cost and share of costs of injectables across AWaRe groups—public and private sectors, Kerala state, India.

AWaRe groups	Public sector cost in *million Indian Rupees* (₹), %	Private sector cost in *million Indian Rupees* (₹), %	Total
Access	₹ 27.4, 9.3%	₹ 266.7, 90.7%	₹ 294.1
Watch	₹ 111.0, 6.9%	₹ 1,493.1, 93.1%	₹ 1,604.1
Reserve	₹ 4.0, 2.4%	₹ 163.0, 97.6%	₹ 167.0
Discouraged	₹ 25.0, 4.1%	₹ 582.5, 95.9%	₹ 607.5
Total	₹ 167.4, 6.3%	₹ 2,505.3, 93.7%	₹ 2,672.7

In ATC 3 level, cephalosporins (and other beta-lactam antibacterials) were the most consumed, of which 32.0% were provided by the public sector at only 4.9% of the total cost spent on this group of medicines. ( Table 10) Combinations of antibiotics were used only in the private sector, and 25.6% of total quinolones came from the public sector. Public sector units in Kerala utilized a higher volume of beta-lactam penicillin injections —20.5% compared to 13.7% in private sector units – and spent about 39.7% of their budget on penicillin which is much higher compared to 14.7% in private spending ( Table 11, Table 12). Other beta-lactam antibacterials (which mainly includes cephalosporins) are used in similar proportions across both sectors— 59.8% in private and 61.3% in public— albeit with different shares of the cost: 66.2% of total spending on injectables in the private sector goes to these medicines compared to only 50.6% of spending in the public sector.

**Table 10.** Share of volume and costs of injectables across ATC 3 groups—public and private sectors, Kerala state, India.

ATC 3 groups	Total DDDs *(’000s)*	DDD share private, %	DDD share public, %	Cost share, private, %	Cost share, public, %
Other beta-lactam antibacterials	4,916.0	68.0%	32.0%	95.1%	4.9%
Beta-lactam antibacterials, penicillin	1,290.9	59.2%	40.8%	84.7%	15.3%
Aminoglycoside antibacterials	1,129.4	76.0%	24.0%	96.6%	3.4%
Quinolone antibacterials	464.4	74.4%	25.6%	93.5%	6.5%
Other antibacterials	156.4	70.5%	29.5%	97.2%	2.8%
Combinations of antibacterials	100.8	100.0%	0.0%	100.0%	0.0%
Macrolides, lincosamides and streptogramins	96.0	70.1%	29.9%	96.7%	3.3%
Tetracyclines	4.8	95.0%	5.0%	98.3%	1.7%
Amphenicols	0.05	100.0%	0.0%	100.0%	0.0%

Note: ATC 3 groups are shown in decreasing order of total consumption (DDDs, ‘000s)

**Table 11.** Share of antibiotic classes (ATC 3) in private sector, Kerala, India, 2016–19.

ATC 3	Share of DDD, %	Share of cost, %
Other beta-lactam antibacterials	59.8%	66.2%
Beta-lactam antibacterials, penicillin	13.7%	14.7%
Other antibacterials	2.0%	9.4%
Aminoglycoside antibacterials	15.3%	4.2%
Quinolones	6.2%	2.5%
Macrolides, lincosamides and streptogramins	1.2%	1.5%
Combinations of antibacterials	1.8%	1.1%
Tetracyclines	0.1%	0.5%
Amphenicols	0.001%	0.0%

**Table 12.** Share of antibiotic classes (ATC 3) in public sector, Kerala, India, 2016–19.

ATC 3	Share of DDDs, %	Share of cost, %
Other beta-lactam antibacterials	61.3%	50.6%
Beta-lactam antibacterials, penicillin	20.5%	39.7%
Other antibacterials	1.8%	4.0%
Quinolones	4.6%	2.5%
Aminoglycoside antibacterials	10.6%	2.2%
Macrolides, lincosamides and streptogramins	1.1%	0.8%
Tetracyclines	0.01%	0.1%

At the ATC 4 groups ( Table 13– Table 15), the public sector used 30.4% of its total antibiotic spending on penicillin combinations, compared to 14.0% of the total spending in the private sector. Carbapenems constituted 2.3% of total DDDs used in the private sector and 0.5% in the public sector. However, they accounted for 24.4% of total spending in the private sector compared to 2.7% of total spending in the public sector.

**Table 13.** Share of volume and costs of injectables across ATC 4 subgroups—public and private sectors, Kerala, India, 2016-19.

ATC 4	DDD share private, %	DDD share public, %	Cost share, private, %	Cost share, public, %
Third generation cephalosporins	67.4%	32.6%	92.5%	7.5%
Other aminoglycosides	76.0%	24.0%	96.6%	3.4%
Combinations of penicillin, incl. BLI	72.2%	27.8%	87.1%	12.9%
Fluoroquinolones	74.4%	25.6%	93.5%	6.5%
Second-generation cephalosporins	63.1%	36.9%	94.7%	5.3%
Beta-lactamase sensitive penicillin	2.4%	97.6%	2.2%	97.8%
Penicillin with extended spectrum	45.1%	54.9%	73.5%	26.5%
Carbapenems	90.9%	9.1%	99.2%	0.8%
Combinations of antibacterials	100.0%	0.0%	100.0%	0.0%
Other antibacterials	71.2%	28.8%	95.1%	4.9%

Note: BLI—Beta-lactamase inhibitors; Only the top ten ATC 4 subgroups (in terms of total DDDs) shown in this table. The full list is given at the end of the document.

**Table 14.** Share of antibiotic classes (ATC 4) in public sector, Kerala, 2016–19.

ATC 4	Share of DDD, %	Share of cost, %
Third generation cephalosporins	55.7%	42.3%
Other aminoglycosides	10.6%	2.2%
Combinations of penicillin, incl. BLI	10.4%	30.4%
Beta-lactamase sensitive penicillin	6.3%	4.9%
Second-generation cephalosporins	5.1%	5.1%

Note: BLI—Beta-lactamase inhibitors

**Table 15.** Share of Antibiotic classes (ATC 4) in private sector, Kerala, 2016–19.

*ATC 4 Class*	Share of DDDs, %	Share of cost, %
Third generation cephalosporins	52.8%	35.5%
Other aminoglycosides	15.3%	4.2%
Combinations of penicillin, incl. BLI	12.4%	14.0%
Fluroquinolones	6.2%	2.5%
Second – generation cephalosporins	4.0%	6.1%

Note: BLI—Beta-lactamase inhibitors

There were 21 injectable antibiotic formulations used in the public sector compared to 69 in the private. ( Table 16– Table 18) In the public sector, the top used injectable —in DDDs— was cefotaxime (26.8% DDDs and 20.3% of cost), while in terms of total cost, piperacillin—tazobactam combination accounted for 25.0% of total spending (6.6% DDDs). In the private sector, meropenem had the highest cost share at formulation level—22.8% of total spending, despite accounting for just 2.1% of DDDs.

**Table 16.** Share of antibiotic group ATC 5 (top 5) in public sector, Kerala, 2016–19.

ATC 5	Share of DDDs, %	Share of cost, %
Cefotaxime	26.8%	20.3%
Ceftriaxone	18.4%	7.1%
Gentamicin	10.5%	2.2%
Cefoperazone - sulbactam	10.3%	14.7%
Piperacillin + tazobactam	6.6%	25.0%

**Table 17.** Share of antibiotic group ATC 5 (top 5) in private sector, Kerala, 2016–19.

ATC 5	Share of DDDs, %	Share of cost, %
Ceftriaxone	31.4%	8.9%
Cefotaxime	9.5%	3.4%
Gentamicin	7.8%	0.49%
Amikacin	7.5%	3.5%
Ceftriaxone - sulbactam	5.9%	3.9%

**Table 18.** ATC5 - injectable antibiotics in public and private sector.

Molecules	Private DDD, (‘000)	Public DDD, (‘000)	Total DDD, (‘000)	Private DDD, %	Public DDD, (%)	Private Cost, (‘000 ₹)	Public Cost, (‘000 ₹)	Total Cost, (‘000 ₹)	Cost, Private, %	Cost, Public, %
Amoxicillin - clavulanic acid	239.6	95.5	335.1	71.5%	28.5%	114,490.3	8,974.1	123,464.3	92.7%	7.3%
Ampicillin	58.9	79.6	138.5	42.5%	57.5%	13,160.9	5,616.0	18,776.9	70.1%	29.9%
Azithromycin	28.7	23.0	51.7	55.6%	44.4%	4,873.8	1,292.7	6,166.5	79.0%	21.0%
Aztreonam	0.6	1.4	2.0	31.3%	68.7%	1,905.0	2,168.1	4,073.1	46.8%	53.2%
Benzylpenicillin	-	161.4	161.4	0.0%	100.0%	-	8,418.6	8,418.6	0.0%	100.0%
Cefoperazone - sulbactam	186.9	264.6	451.5	41.4%	58.6%	392,464.6	25,035.1	417,499.6	94.0%	6.0%
Cefotaxime	531.8	691.7	1223.5	43.5%	56.5%	84,675.9	34,730.6	119,406.5	70.9%	29.1%
Ceftazidime	13.5	2.1	15.6	86.6%	13.4%	11,609.5	336.2	11,945.6	97.2%	2.8%
Ceftriaxone	1754.7	475.0	2229.7	78.7%	21.3%	224,424.1	12,097.3	236,521.4	94.9%	5.1%
Cefuroxime	223.3	130.8	354.1	63.1%	36.9%	152,998.8	8,634.9	161,633.7	94.7%	5.3%
Ciprofloxacin	77.6	94.2	171.8	45.2%	54.8%	6,025.8	3,638.3	9,664.1	62.4%	37.6%
Cloxacillin	-	19.1	19.1	0.0%	100.0%	-	750.5	750.5	0.0%	100.0%
Gentamicin	433.6	271.6	705.2	61.5%	38.5%	12,327.4	3,687.3	16,014.6	77.0%	23.0%
Levofloxacin	140.5	13.2	153.8	91.4%	8.6%	22,153.6	370.7	22,524.3	98.4%	1.6%
Linezolid	58.7	23.8	82.5	71.2%	28.8%	63,933.0	3,317.0	67,250.0	95.1%	4.9%
Meropenem	120.0	13.0	133.0	90.2%	9.8%	570,866.8	4,662.4	575,529.2	99.2%	0.8%
Ofloxacin	107.8	21.3	129.0	83.5%	16.5%	27,120.6	539.8	27,660.4	98.0%	2.0%
Piperacillin -tazobactam	196.1	170.9	367.0	53.4%	46.6%	224,435.3	42,797.5	267,232.7	84.0%	16.0%
Teicoplanin	33.4	1.0	34.4	97.2%	2.8%	78,317.0	327.0	78,644.0	99.6%	0.4%
Tigecycline	1.9	0.2	2.1	88.6%	11.4%	11,584.9	237.2	11,822.0	98.0%	2.0%
Vancomycin	10.1	21.7	31.9	31.8%	68.2%	11,205.8	3,222.1	14,428.0	77.7%	22.3%

Note: only those antibiotics provided in public sector included in this table; all costs are in Indian Rupees (₹)

## Discussion and conclusion

This is the first study from India that estimated the total and the public and private share of antibiotic consumption using WHO DDD methodology. The study used large scale private sector sales data and public sector (Kerala state) procurement data to examine the share of antibiotic volume and cost by WHO AWaRe and ATC classifications, thus providing some indications on the level of appropriateness of antibiotic use. The study showed the importance of injectable antibiotics as an area of intervention for governments to improve access and appropriate use of antibiotics.

Oral preparations constituted the majority of antibiotics used, likely because they are more readily available, are easily consumable, and are the first choice of treatment at the primary and secondary level of the healthcare system. In comparison, injectables are used only in in-patient care and are harder to administer. However, the relatively higher share of cost for injectables make them an important target for interventions in improving access.

Watch group antibiotics were significantly more consumed and at a significantly higher cost than Access group antibiotics. While Reserve group antibiotics make up the lowest consumption share, their cost per DDD is the highest. The Discouraged group of antibiotics shows high consumption (21.9%) and high share of cost (26.3%). This raises the question of how much antibiotic stewardship can be achieved through drug price control at the policy and regulatory level. Expensive, later-line molecules like ceftriaxone and cefotaxime had a higher share of consumption in both sectors in Kerala, despite cheaper alternatives such as penicillin, amikacin, and gentamicin. This could either signify a lack of antimicrobial stewardship, a rampant spread of antimicrobial resistant diseases that require more expensive injectable antimicrobial therapy, patient expectations, or financial incentives to prescribe costly antibiotics ^15, 16^.

The private sector consumption patterns in ATC 3 level at the national and state level were similar, with three notable exceptions. Firstly, the private sector in Kerala uses less aminoglycosides— 15.3% of the total injectables compared to the national average of 22.7%; secondly, the Kerala private sector uses more quinolones compared to the national average— 6.2% vs 3.2%; and thirdly, Kerala uses less macrolides, lincosamides, and streptogramins (1.2%) compared to the national average of 3.6%. The per-dose cost varies widely between sectors— penicillin costs INR 126.4 per dose in the public sector compared to INR 480.6 in the private sector; other beta-lactam antibacterials (cephalosporins and others) cost INR 53.8 in public compared to INR 496.0 in private; and the J01X- “other antibacterials” costs INR 146.9 per dose in public and INR 2133.3 per dose in private.

This study corrects a common assumption that while a large proportion of oral community use antibiotics are dispensed at private pharmacies and hospitals on an OOP basis, this proportion is lower for injectable antibiotics. Using national data from India and private sector consumption data from Kerala state, this study shows that the share of private sector consumption is equally high for injectable antibiotics (~68%). In the private sector, over 33% of the total spending on antibiotics is for injectables, while in the public sector, this proportion is slightly over 25%. Across all groups of antibiotics, the cost per DDD is significantly higher in the private sector as compared to the cost per DDD in the public sector. At both the national and Kerala state levels, the consumption patterns across different antibiotics classes were similar in the private sector, with some notable exceptions.

This study serves as a reminder that antibiotic stewardship initiatives must not overlook the significance of private pharmacies and private hospitals as essential sources of antibiotic supply, including injectable antibiotics. Effectively managing this aspect is crucial from both the perspective of combating Antimicrobial Resistance (AMR) and ensuring access, cost-effectiveness, and efficiency within the health system.

## Data Availability

The PharmaTrac data used in this study are available from the AIOCD Pharma soft tech AWACS Pvt. Ltd. Restrictions apply to the availability of these data, which were used under license for this study. Permission can be obtained at https://aiocdawacs.com. All analysis were conducted using R software.
