# Supplementary material for: Injectable antibiotic use in India: public-private share in volume and cost
Source: Wellcome Open Res. 2024 Feb 19;9:70. [Version 1] doi: 10.12688/wellcomeopenres.20633.1 (PMC11362719; doi:10.12688/wellcomeopenres.20633.1)
Supplement: Peer review history

# Reviewer report: Reviewer response for version 1

**DOI:** 10.21956/wellcomeopenres.22836.r77845

**Published:** 2024-08-29

**Authors:** Idemudia Imonikhe Otaigbe

**Affiliations:** 1 Department of Medical Microbiology, School of Basic Clinical Sciences, Benjamin Carson (Snr) College of Health and Medical Sciences, Babcock University/Babcock University Teaching Hospital, Ilishan-Remo, Ogun State, Nigeria

"Injectable antibiotic use in India: public-private share in volume and cost"

**SUMMARY**

 This is a very interesting study and the authors have done significant justice to the topic. The study objectives are clearly stated and the methodology is well described. However, to improve the quality of the article, I have outlined some comments below.  

**Comment 1**

 The authors should clearly justify or explain the following: 

 1. How their study will improve the appropriate use of injectable antibiotics in the state of Kerala and India.

 2. Why they chose the state of Kerala.

**Comment 2 (Abstract)**

 Consumption of injectable antibiotics is not widely studied, despite injectables constitute a major share of antibiotic cost.

 Please correct to: “Consumption of injectable antibiotics is not widely studied, despite injectables constituting a major share of antibiotic cost.”

**Comment 3 (Objectives)**

 Your objectives are germane. However did you consider capturing the volume of injectables used in both outpatient and in-patient settings in the public and private sector? Do you have data which shows how many injectables were given in out-patient and in-patient settings. This is important since in some low and middle income countries patients in out-patient settings may have strong cultural preferences for injectable antibiotics (injectable antibiotics are deemed to be ‘stronger’ or more effective than oral antibiotics) even when oral antibiotics are suitable and prescribed.

**Comment 4 (Methods)**

 Please provide a citation for the following definition ……. **Defined Daily Dose (DDD), which is the assumed average maintenance dose per day used for the main indication in adults for a drug based on its ATC groups.**

 Please provide citations and references for how you calculated the following:

  -Total DDDs consumed

 - DDD per 1000 persons per day,

 Please provide a citation for the following definition **“MRP refers to the maximum price at which a product can be sold to the consumer in the private sector pharmacies or hospitals after including the profit margins, and therefore represents the cost incurred by the patients treated at the private sector at the point of care.”**

 Please provide a citation for the following sentence **“Wholesale price refers to MRP minus the profit margin for pharmacies or hospitals, applying wholesale prices will make the private sector purchase prices closer to public sector procurement price.”**

 Please provide the full meanings of these abbreviations if possible: 

 AIOCD

 AWACS                 

 More than 33% of total spending in private sector is for injectables, compared to a little over 25% in public sector (Table 7).

 Please include the word “the”  before private sector. The sentence thus reads:

 More than 33% of total spending in the private sector is for injectables, compared to a little over 25% in public sector (Table 7).

 What accounts for the larger spending in the private sector on injectables? Patient preferences? Guidelines? More revenue generated from injectables compared to oral antibiotics?

**Comment 5**

 Was ethical approval obtained for this study? Please mention the source of ethical approval.

**Comment 6 (Discussion and conclusion** )

 I suggest separating the discussion section of the paper from the conclusion. 

 In your discussion please compare your results with previous studies, particularly in low and middle income countries. For example these studies may be of help: 

- Ofori-Asenso R,et al. (2015 ^1^)
- Mohlala G, et al. (2010 ^2^)
- Wirtz VJ, et al. (2010 ^3^)
- Ahiabu MA, et al. (2016 ^4^)
- Skosana PP et al. (2022 ^5^)

Is the work clearly and accurately presented and does it cite the current literature?

Yes

If applicable, is the statistical analysis and its interpretation appropriate?

Yes

Are all the source data underlying the results available to ensure full reproducibility?

Partly

Is the study design appropriate and is the work technically sound?

Yes

Are the conclusions drawn adequately supported by the results?

Yes

Are sufficient details of methods and analysis provided to allow replication by others?

Yes

Reviewer Expertise:

I am a Consultant Clinical Microbiologist. My research interests include antimicrobial resistance, antimicrobial stewardship and infection prevention and control.

I confirm that I have read this submission and believe that I have an appropriate level of expertise to confirm that it is of an acceptable scientific standard.

## References

[1] : A review of injection and antibiotic use at primary health care (public and private) centers in Africa. *J Pharm Bioallied Sci* .2015;7(3) : 10.4103/0975-7406.160008 175-80 10.4103/0975-7406.160008 26229350 PMC4517318

[2] : Drug prescription habits in public and private health facilities in 2 provinces in South Africa. *East Mediterr Health J* .2010;16(3) :324-8 20795449

[3] : Trends in antibiotic utilization in eight Latin American countries, 1997-2007. *Rev Panam Salud Publica* .2010;27(3) : 10.1590/s1020-49892010000300009 219-25 10.1590/s1020-49892010000300009 20414511

[4] : A retrospective audit of antibiotic prescriptions in primary health-care facilities in Eastern Region, Ghana. *Health Policy Plan* .2016;31(2) : 10.1093/heapol/czv048 250-8 10.1093/heapol/czv048 26045328 PMC4748131

[5] : A national, multicentre web-based point prevalence survey of antimicrobial use in community healthcare centres across South Africa and the implications. *Hosp Pract (1995)* .2022;50(4) : 10.1080/21548331.2022.2114251 306-317 10.1080/21548331.2022.2114251 35980901

# Reviewer report: Reviewer response for version 1

**DOI:** 10.21956/wellcomeopenres.22836.r85371

**Published:** 2024-06-18

**Authors:** Mathew Hennessey

**Affiliations:** 1 The Royal Veterinary College, London, UK

The study is an interesting concept and aims to address questions about antibiotic consumption at the national/state level, which has been lacking from India. However, the article is currently lacking focus on key results and a 'so what' making it difficult to interpret the results. 

 Overall the introduction needs to refer to existing literature more, particularly around how inappropriate antibiotic use is defined. This is necessary to understand what the ideal situation would look like for such a dataset. There are currently many tables in the paper and I would suggest reducing these to those which show your key findings, and then moving some to an appendix (so have around 6 max in the paper). At the moment the written results are mostly a repetition of the tables and it would be better for text to talk about key findings with some subheadings to signpost the reader. Similarly, the discussion mostly repeats the results and does not situate key findings within the wider literature.

 Introduction

- Bacteria names should be in italics
- Para 2 – You say India faces serious challenges related to inappropriate use of antibiotics – but do not give any examples, or indicate how you define inappropriate use in this context
- Para 3 – At the end when you talk about lack of access – are you referring to injectables here, or W and R Abs? At the moment it is unclear.
- Para 4 – Needs some clarity. You mention the direct and indirect costs are not limited to death and disability. Is it your intention here to focus just on human health costs (rather than also talking about the costs in animal health and food production)? If so then I think you should make this clear.  You then provide one other example of costs associated with AMR (prolonged costs of treatment). Is it your intention here to suggest this is the only other cost, or that this is one of several possible other costs? You then go on to say, “Therefore, existing health inequities complicate the problem in the Indian context”. What do you mean by this sentence? You then mention differences in urban and rural access and funding for the public sector. Can you elaborate on why and how these factors impact the costs of AMR? At the moment this is unclear
- Para 6 – talks about literature on ABU patterns, but does not cite any.
- Para 7 – why are payment and procurement models needed to mitigate the risks of AMR? I’m not saying they do not, but where is the argument or evidence for this? This needs to be backed up with literature. 

Results 

 National level consumption

- I found the first table confusing, I think it would be better to present the DDDs consumed and cost per 1000 population as you have done in the text (and then have consistency between all the tables). 
- Initially when you talk about national Abs you do not differentiate total oral vs inj by private and public – it would be good to see this, and then be able to see later how Kerala compares to the national data.
- You talk about ‘discouraged’ Abs in reference to table 2, but this is not introduced in the intro, how is discouraged defined? Is this combination antibiotics? If so, this needs to be explained.

Kerala

- I think this section would benefit from a reduction in tables, focusing on key results within the text, and subheadings to signpost the reader and allow some comparison to national figures where possible 

Discussion 

 Generally, the discussion is too much a repeat of the results. It needs to highlight the key results briefly and then talk about the relevance of these to help the reader understand the 'so what' and to think about what an ideal pattern of antibiotic use would look like in terms of oral vs injectable and public vs private provision of treatment. 

- At the start of the discussion you say the study provides an indication of appropriateness, how? I think to make this claim you need to provide some indication in the introduction of what you would expect the shares of injectable vs oral, and shares between different AWR Abs. Then you would be able to compare you findings to a benchmark and comment on appropriateness. At the moment, as you do not clearly define ‘appropriateness’ in the introduction.
  - You say the study shows importance of injectable as an area for intervention – why? What does the literature say about how injectables are used in the informal sector? 

Kerala is one of the only Indian states to have its own State Action Plan on AMR, is there anything in this which relates to your study? 

 I look forward to seeing a revised version of this paper.

Is the work clearly and accurately presented and does it cite the current literature?

No

If applicable, is the statistical analysis and its interpretation appropriate?

Not applicable

Are all the source data underlying the results available to ensure full reproducibility?

No

Is the study design appropriate and is the work technically sound?

Yes

Are the conclusions drawn adequately supported by the results?

No

Are sufficient details of methods and analysis provided to allow replication by others?

Yes

Reviewer Expertise:

Antibiotic use decisions in livestock in India

I confirm that I have read this submission and believe that I have an appropriate level of expertise to confirm that it is of an acceptable scientific standard, however I have significant reservations, as outlined above.

# Reviewer report: Reviewer response for version 1

**DOI:** 10.21956/wellcomeopenres.22836.r85365

**Published:** 2024-06-12

**Authors:** Nam Nguyen

**Affiliations:** 1 University of Oxford, Oxford, UK

1. In general, the article will need to be substantially revised before being resubmitted.  

 2. The work should be presented in a more constructive way.

 - In the background section, there is a lack of systematic information on the demographic context and health systems (both in India and in Kerala). I.e. what types of health facilities are included in the public and private health systems (along with specific numbers). Are the proportions of primary care facilities and the case mix of patients very different between them, making comparisons inappropriate?

 - Critical information missing from the method (see below)

 - There are a total of 18 tables in the results (which should normally be limited to about 5). Many of these can be combined (e.g. 11&12 and 14&15) or presented as figures, especially if the main purpose is to compare between groups of antibiotics or the distribution of each group between the public and private sectors.

 - Combining tables/figures with similar messages is also useful to focus findings. For figures that are not really relevant to the discussion, the author may consider keeping them in supplementary documents.

 - Lack of discussion of the limitations of the study

 - The main conclusion that the public sector showed higher cost efficiency in antibiotic provisioning compared to the private sector is not appropriate. The aim of the study is only to estimate the volume and provide a rough comparison between these sectors. If the differences in patient case mix between these sectors are not taken into account and adjusted for, the lower consumption of antibiotics cannot reflect the differences in efficiency.

 3. Details of methods and analyses provided are insufficient to allow replication:

 - Study design not reported

 - Data source (PharmaTrac & KMSCL) not described in detail (managed by whom, who reported and to whom reported [by regulation or voluntarily], which modes of settings [with number if available, or if not, needed a further discussion on limitation] included, mechanisms used to ensure reliability and validity, previous use of the data source for research purposes).

 - How was the DDD determined for fixed-dose combination products for which the DDD is not available in the WHO ATC/DDD Index 2019 list (if available)?

 - How was the cost efficiency in injectable antibiotic provisioning (mentioned in the conclusion) defined and measured, taking into account differences in patient case mix between public and private health care settings?

  - Cost discounting should be applied as the study covers a rather long period 2016-2019.

 4. Comparison in antibiotic use between Kerala and national level should be performed and discussed

 5. Comparison of antibiotic use between Kerala and national level should be made and discussed.

 6. The statistical analysis is appropriate for the purpose of the study, but the interpretation is not appropriate. The main conclusion is over-interpreted (see comments above).

 7. The conclusions drawn is inadequately supported by the results, see comment above

Is the work clearly and accurately presented and does it cite the current literature?

No

If applicable, is the statistical analysis and its interpretation appropriate?

Partly

Are all the source data underlying the results available to ensure full reproducibility?

No

Is the study design appropriate and is the work technically sound?

Partly

Are the conclusions drawn adequately supported by the results?

No

Are sufficient details of methods and analysis provided to allow replication by others?

No

Reviewer Expertise:

pharmacoepidemiology, health economics, pharmacy practice

I confirm that I have read this submission and believe that I have an appropriate level of expertise to confirm that it is of an acceptable scientific standard, however I have significant reservations, as outlined above.
